# Cellular and genetic models of H6PDH and 11β‐HSD1 function in skeletal muscle

**DOI:** 10.1002/cbf.3272

**Published:** 2017-07-27

**Authors:** Agnieszka E. Zielinska, Rachel S. Fletcher, Mark Sherlock, Craig L. Doig, Gareth G. Lavery

**Affiliations:** ^1^ Institute of Metabolism and Systems Research University of Birmingham Birmingham UK; ^2^ Centre for Endocrinology, Diabetes and Metabolism Birmingham Health Partners Birmingham UK

**Keywords:** 11β‐HSD1, G6PT, glucocorticoids, H6PDH, skeletal muscle

## Abstract

Glucocorticoids are important for skeletal muscle energy metabolism, regulating glucose utilization, insulin sensitivity, and muscle mass. Nicotinamide adenine dinucleotide phosphate‐dependent 11β‐hydroxysteroid dehydrogenase type 1 (11β‐HSD1)‐mediated glucocorticoid activation in the sarcoplasmic reticulum (SR) is integral to mediating the detrimental effects of glucocorticoid excess in muscle. 11β‐Hydroxysteroid dehydrogenase type 1 activity requires glucose‐6‐phosphate transporter (G6PT)‐mediated G6P transport into the SR for its metabolism by hexose‐6‐phosphate dehydrogenase (H6PDH) for NADPH generation. Here, we examine the G6PT/H6PDH/11β‐HSD1 triad in differentiating myotubes and explore the consequences of muscle‐specific knockout of 11β‐HSD1 and H6PDH. 11β‐Hydroxysteroid dehydrogenase type 1 expression and activity increase with myotube differentiation and in response to glucocorticoids. Hexose‐6‐phosphate dehydrogenase shows some elevation in expression with differentiation and in response to glucocorticoid, while G6PT appears largely unresponsive to these particular conditions. When examining 11β‐HSD1 muscle‐knockout mice, we were unable to detect significant decrements in activity, despite using a well‐validated muscle‐specific Cre transgene and confirming high‐level recombination of the floxed HSD11B1 allele. We propose that the level of recombination at the HSD11B1 locus may be insufficient to negate basal 11β‐HSD1 activity for a protein with a long half‐life. Hexose‐6‐phosphate dehydrogenase was undetectable in H6PDH muscle‐knockout mice, which display the myopathic phenotype seen in global KO mice, validating the importance of SR NADPH generation. We envisage these data and models finding utility when investigating the muscle‐specific functions of the 11β‐HSD1/G6PT/H6PDH triad.

## 1 INTRODUCTION

Glucocorticoids fulfil important permissive and adaptive roles in the regulation of skeletal muscle energy metabolism, impacting glucose utilization, insulin sensitivity, amino acid, and lipid metabolism.^1^ Glucocorticoids can also influence muscle mass by regulating the balance between pathways affecting protein synthesis and protein degradation, via modulation of amino acid transport, the anabolic actions of insulin and IGF1, and through the regulation of catabolic pathways such as the ubiquitin proteasome system and E3 ubiquitin ligases atrogin‐1 and muscle ring finger 1.^2^, ^3^ Indeed, the importance of glucocorticoids to muscle is exemplified in patients with glucocorticoid excess (Cushing's syndrome) who develop insulin resistance, muscle weakness, and profound proximal myopathy.^4^

Glucocorticoid availability in muscle depends on circulating delivery and intracellular activation by 11β‐hydroxysteroid dehydrogenase type 1 (11β‐HSD1), which, in rodents, converts inactive 11‐dehydrocorticosterone (11‐DHC) to corticosterone in a nicotinamide adenine dinucleotide phosphate (NADPH)‐dependent reaction.^5^ 11β‐Hydroxysteroid dehydrogenase type 1 activity is dependent upon the maintenance of an appropriate NADPH/NADP+ ratio at its site of activity within the lumen of the sarcoplasmic reticulum (SR).^6^, ^7^, ^8^ To achieve this, glucose‐6‐phosphate (G6P) is transported into the SR by the G6P transporter (G6PT) and metabolized by the enzyme hexose‐6‐phosphate dehydrogenase (H6PDH) to produce 6‐phosphogluconate and NADPH.^9^ In H6PDH knockout (H6MKO) mice, a lack of adequate NADPH generation and disruption of the H6PDH/11β‐HSD1 interaction result in 11β‐HSD1 assuming activity to inactivate glucocorticoids and increase their clearance.^9^, ^10^, ^11^

The role of 11β‐HSD1 in regulating muscle‐specific insulin signalling and lipid utilization has been established.^12^, ^13^ 11β‐Hydroxysteroid dehydrogenase type 1 expression and activity are elevated in type 2 diabetic muscles, and so 11β‐HSD1 inhibition may offer potential to enhance insulin sensitivity.^12^ However, not all 11β‐HSD1 knockout (HSD1KO) loss‐of‐function models have demonstrated protection from metabolic disease and suggest that use of inhibitors will need to be tailored to specific pathologies.^14^, ^15^ Indeed, it has been shown that 11β‐HSD1KO mice are almost completely protected from the deleterious metabolic effects of glucocorticoid excess, and inhibition in this scenario may be truly beneficial.^3^, ^12^, ^13^, ^16^

To better evaluate the muscle‐specific contributions of 11β‐HSD1 and H6PDH to local and global glucocorticoid‐regulated metabolic homeostasis, we have evaluated myotube expression and regulation of the intraluminal enzymes 11β‐HSD1/H6PDH/G6PT and generated skeletal muscle‐specific knockouts of 11β‐HSD1 (HSD1MKO) and H6PDH (H6MKO). It is envisaged that these data will permit a more thorough appreciation of their muscle‐specific functions in the context of glucocorticoid excess and metabolic disease.

## 2 MATERIALS AND METHODS

Unless stated otherwise, all materials and reagents were purchased from Sigma‐Aldrich, UK.

### 2.1 Cell culture

The mouse myoblast cell line, C2C12, is a well‐established model of both skeletal muscle proliferation and differentiation. Proliferating C2C12 myoblasts were cultured in Dulbecco's Modified Eagle Medium (DMEM) supplemented with 10% fetal calf serum and seeded into 12‐well tissue culture plates. Differentiation was initiated when cells reached 60 to 70% confluence by replacing proliferation media with DMEM supplemented with 5% horse serum and carried on for 8 days. Prior to treatment, cells were incubated in serum‐free DMEM for 4 hours. Primary myoblasts were generated from isolated satellite cells of the mouse extensor digitorum longus muscle. Primary myoblasts were maintained in 12‐well tissue culture plates in 1 mL of DMEM supplemented with 10% horse serum and 0.5% chick embryo extract. Differentiation into myotubes was initiated by replacing proliferating medium with DMEM supplemented with 2% horse serum and 0.5% chick embryo extract after myoblasts reached 60 to 70% confluence. Differentiation medium was replaced every 48 hours. After 8 days of differentiation, myoblasts fused to form multinucleated myotubes. Cells were treated with dexamethasone (DEX; 1 μM), insulin (1 μM), glucocorticoid receptor antagonist, Ru38486 (5 μM), and combination of DEX with insulin and DEX with Ru38486. In experiments using Ru38486, cells were pretreated with Ru38486 for 10 minutes before adding DEX in DMEM media. Cell treatments were carried out for 24 hours.

### 2.2 Animal generation and maintenance

All studies were conducted on male HSD1MKO, H6MKO, and wild‐type (WT) mice group‐housed under controlled temperature (21‐23°C) and light (12 h light, 12 h dark cycle; lights on at 0700 h). The mice had ad libitum access to water and standard chow. Animal procedures were approved under the British Home Office Animals (Scientific Procedures) Act 1986 and through the Local Animal Ethics Committee.

### 2.3 Muscle‐specific 11β‐HSD1 knockout mouse model

Previously, a conditional *HSD11B1* allele was generated by flanking exon 5 with LoxP sites, and from this, global 11β‐HSD1KO were derived.^17^ To generate mice devoid of 11β‐HSD1 activity specifically in skeletal muscle, we crossed homozygous floxed *HSD11B1* mice on a mixed C57BL/6J/129SvJ background with 3 different skeletal muscle specific‐Cre transgenic mice: MEF2c‐Cre, ACTA1‐Cre, and MCK‐Cre (Jackson Labs, all C57BL/6J). Each transgenic restricts Cre expression to skeletal muscle tissue and to a lesser degree heart. Genotyping PCR was carried out on ear clip DNA by using gene specific primers (5′‐3′) P1‐GGGAGCTTGCTTACAGCATC, P2‐CATTCTCAAGGTAGATTGAACTCTG, and P3‐TCCATGCAATCAACTTCTCG. P1 + P2 give a 138 bp product for a WT allele and a 172 bp product for a conditional allele containing a 3′ LoxP site (Lox+/+). After a P2 binding site is removed and P1 + P3 are brought into proximity, they produce a 279 bp KO band confirming successful Cre recombination and exon 5 removal.

### 2.4 H6PDH muscle‐knockout mouse model

The H6MKO first allele mice were purchased from the European Conditional Mouse Mutagenesis Program (the European Mouse Mutant Archive‐EMMA, Munich, Germany) that employs a KO first allele strategy in which an SA‐βgeo‐pA reporter cassette (SA, splice acceptor; βgeo, β‐galactosidase/neomycin phosphotransferase fusion gene; pA, bovine growth hormone polyadenylation sequence) flanked by flippase recognition target sequence is inserted into the first intron of H6PDH inactivating the gene. This global knockout was called H6KO. A conditional allele was generated by Flp recombinase expression to remove the reporter gene. Cre recombinase expression under the control of the *Acta1* promoter excised exon 3 coding the catalytic domain of the conditional *H6PD* allele in skeletal muscle. To generate H6MKO, floxed homozygous H6KO mice on a C57BL/6 background were crossed with Acta1‐Cre transgenic mice (C57BL/6 background, targeting Cre expression to skeletal muscle and heart). This generated mice devoid of H6PDH activity in skeletal muscle cells. Genotyping PCR was carried out on ear clip DNA by using the following gene specific primers (5′‐3′) Ef4685‐TTTGCACGGGCCTCAGGGTGG, L3r4688‐TGGCTTTGGGAGGGAGTGGCCC, CreF‐GTAGTTATTCGGATCATCAGCTACAC, and CreR‐GCTGCCACGACCAAGTGACAGCAATG. The Ef4685 and L3r4688 primers were used to confirm successful deletion of exon 3, which inactivated *H6PD* function (747 bp), whereas the CreF/R primers confirmed gene knockout specific to skeletal muscle (402 bp).

### 2.5 Western blot analysis

Twenty μg of protein isolated from quadriceps muscle or cells was run on 10% sodium dodecyl sulfate polyacrylamide gel electrophoresis. After electrophoresis, proteins were transferred onto immobilon polyvinyl difluoride membranes (Millipore, Bedford, MA) at 100 V for 1 hour, in a buffer containing 25 mm Tris, 200 mm glycine, and 20% (*v*/v) methanol. The membrane was blocked in phosphate‐buffered saline (PBS) containing 0.1% (*v*/*v*) Tween 20 (PBS‐T) and 5% (*w*/*v*) skimmed milk powder, washed, and then incubated with 11β‐HSD1 (in‐house) or H6PDH (Santa Cruz Biotechnology, Heidelberg, Germany) rabbit polyclonal antibody, diluted 1/1000 overnight at 4°C in PBS‐T. The membrane was then washed and incubated with goat antirabbit secondary antibody diluted 1/25,000 in PBS‐T. Detection was enhanced by chemiluminescence (Amersham Biosciences, Bucks, UK). α‐Tubulin antibody (Santa Cruz Biotechnology, Heidelberg, Germany) was used as a loading control.

### 2.6 11β‐Hydroxysteroid dehydrogenase type 1 activity assay

Tissue explants (~20 mg), 100 μg of muscle microsomes, confluent primary myofibroblasts, or differentiated myotubes were incubated with 100 nM 11‐DHC and 60,000 cpm ^3^H‐11‐DHC. Steroid incubation was carried out for 4 hours for muscle tissue explants, microsomes, and primary cells, while 30 minutes for liver. Subsequently, steroids were extracted by using dichloromethane, separated by using a mobile phase consisting of chloroform/absolute ethanol (92:8) by thin layer chromatography, and scanned by using a Bioscan 200 imaging scanner (LabLogic, Sheffield, UK). Corticosterone and 11‐DHC were purchased from Sigma‐Aldrich (Poole, UK). ^3^H‐Corticosterone (specific activity 1 mCi/mL) was purchased from Amersham Biosciences (Amersham, UK). ^3^H 11‐Dehydrocorticosterone was synthesized “in house” from ^3^H‐corticosterone.

### 2.7 RNA analysis and real‐time polymerase chain reaction

Tissue explants were homogenized with a PowerGen 125 homogenizer (Fisher Scientific, Loughborough, UK). RNA was extracted by using TriReagent (Sigma, Poole, UK) according to the manufacturer's protocol. RNA quality was assessed by 1% agarose gel electrophoresis and quantified spectrophotometrically. Two‐step RT‐PCR was performed by using 1 μg of RNA, random hexamers, and Multiscribe Reverse Transcriptase kit (Life Technologies, Cheshire, UK). Real‐time PCR was carried out as previously described.^17^ Gene expression was assessed by using prevalidated specific TaqMan gene expression assays and Universal PCR Master Mix (Life Technologies). Expression levels were normalized to the housekeeping 18S gene. Data are expressed as arbitrary units by using the following transformation: expression = 1000 × [2^−Δct^]. Fold change in the expression was calculated by using 2^−ΔΔCt^, where ΔCt = (Ct value of gene of interest) − (Ct value of 18S). Data are expressed as fold change with respect to WT animals.

### 2.8 Hexose‐6‐phosphate dehydrogenase activity

Hexose‐6‐phosphate dehydrogenase enzyme activity was measured by spectrophotometric detection of NADPH upon the addition of 10 mM NADP^+^ and 10 mM G6P to 100 μg of skeletal muscle microsomes in a total volume of 300 μL using the Ultrospec 2100pro spectrophotometer (Amersham Biosciences). Microsomes were permeabilized at 4°C with 0.5% Triton X‐100 for 30 minutes to allow the free access of the cofactor to the intraluminal enzyme and incubated in 20 mM MOP buffer (100 mM KCl, 20 mM NaCl, 1 mM MgCl_2_, and pH 7.2) at 37°C. Absorbance readings were taken at 340 nm at 20 second intervals for 3 minutes.

### 2.9 Histological analysis

Quadriceps muscle was harvested from mice and then fixed in 10% neutral buffered formalin. Each muscle from both null and WT mice (*n* = 3 for each genotype) was paraffin embedded and cut into 5 μm sections to be stained by haematoxylin and eosin and by periodic acid‐Schiff according to the manufacturer's protocol for detection of glycogen and to provide histological analysis of muscle structure.

### 2.10 Statistical analysis

Statistical comparisons were performed by using Prism 4 (GraphPad, CA). Data are presented as mean ± SEM with statistical significance defined as *P* less than .05. Where data were normally distributed, unpaired Student *t* tests were used to compare single treatments to control, while one‐way ANOVA on ranks was used to compare multiple treatments or times by using SigmaStat 3.1 (Systat Software, CA, USA). To perform statistical analysis on real‐time PCR data, mean values of delta Ct were used.

## 3 RESULTS

### 3.1 11β‐Hydroxysteroid dehydrogenase type 1 expression and activity increases during muscle cell differentiation

**Figure 1:**
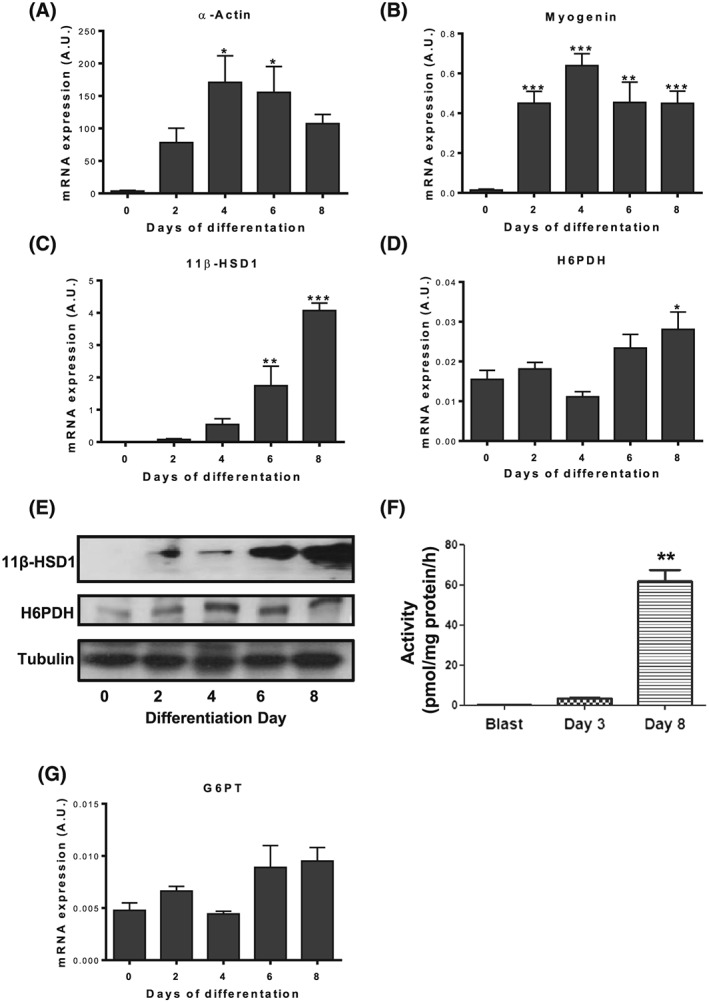
Expression of 11β‐hydroxysteroid dehydrogenase type 1 (11β‐HSD1), hexose‐6‐phosphate dehydrogenase (H6PDH), and glucose‐6‐phosphate transporter (G6PT) in differentiating C2C12 cells The anticipated mRNA expression profile of A, α‐actin and B, myogenin during muscle cell myotube differentiation. C, 11β‐HSD1 and D, H6PDH mRNA expression with E, corresponding protein expression, F, 11β‐HSD1 oxo‐reductase activity, and G, G6PT mRNA expression across differentiation (*n* = 3 in triplicate, *P < .05; **P < .01; ***P < .001 versus day 0 myoblasts)

To explore potential for co‐regulation of 11β‐HSD1/H6PDH/G6PT in muscle, we differentiated C2C12 muscle cells for 8 days, confirming appropriate differentiation according to the upregulated expression of the muscle‐specific markers α‐actin (*P* < .05) and myogenin (*P* < .001; Figure 1A and B). We confirmed previous findings that 11β‐HSD1 mRNA significantly increased during differentiation (*P* < .001; Figure 1C). To endorse this, we measured significantly increased 11β‐HSD1 protein (Figure 1E) and oxo‐reductase activity during differentiation (*P* < .01; Figure 1F). Given the steady increase in 11β‐HSD1 mRNA over differentiation, we measured H6PDH and G6PT mRNA as part of the axis determining 11β‐HSD1 enzyme activity. Hexose‐6‐phosphate dehydrogenase expression was only marginally elevated after 8 days (*P* < .05) not acquiring the same degree of upregulation as 11β‐HSD1 (Figure 1D and E), while there was no significant change in the expression of G6PT (Figure 1G).

### 3.2 Glucocorticoid regulation of 11β‐hydroxysteroid dehydrogenase type 1 and hexose‐6‐phosphate dehydrogenase

**Figure 2:**
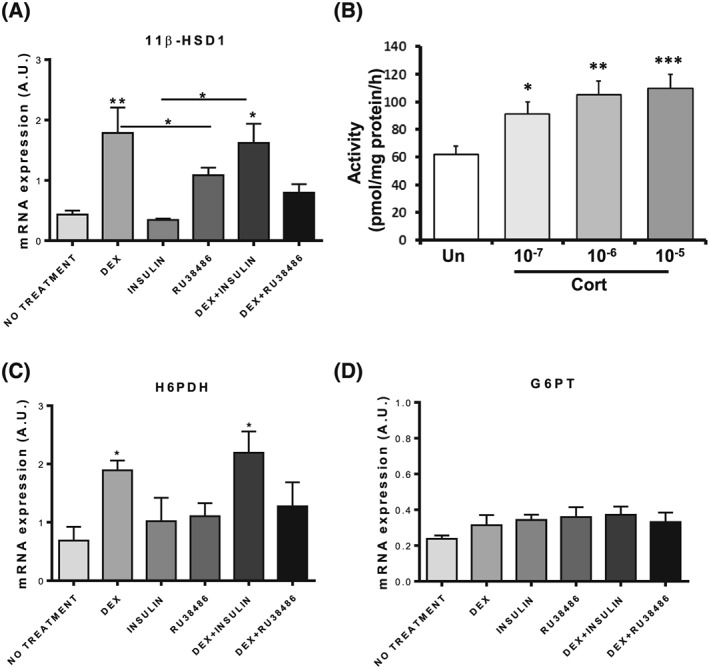
Expression and regulation of 11β‐hydroxysteroid dehydrogenase type 1 (11β‐HSD1), hexose‐6‐phosphate dehydrogenase (H6PDH), and glucose‐6‐phosphate transporter (G6PT) in C2C12 myotubes Following 24 hour treatment with dexamethasone (DEX; 1 μM) with or without glucocorticoid receptor (GR) antagonist Ru38486 (5 μM) or insulin (1 μM) with or without DEX and Ru38486 (A) 11β‐HSD1 mRNA (B) corresponding oxo‐reductase in response to increasing doses of corticosterone. (C) H6PDH mRNA is also responsive to DEX, while (D) G6PT is unresponsive to all conditions (*n* = 3 in triplicate, *P < .05; ** *P* < .01; *** *P* < .001 versus control or relevant treatment)

Besides locomotion, skeletal muscle functions as the body's main storage depot of insulin‐stimulated glucose uptake; therefore, we assessed the 11β‐HSD1/H6PDH/G6PT triad's response to insulin and glucocorticoid exposure. Treatment with synthetic glucocorticoid DEX significantly increased 11β‐HSD1 mRNA expression (*P* < .01). Insulin had no effect and, in combination with DEX, did not oppose increased 11β‐HSD1 expression (*P* < .05). Treatment with the glucocorticoid receptor antagonist Ru38486 prevented DEX‐mediated 11β‐HSD1 upregulation (*P* < .05; Figure 2A). We confirmed that the increased levels of 11β‐HSD1 mRNA were functionally relevant by showing increased reductase activity in cells exposed to increasing concentrations of the endogenous glucocorticoid corticosterone (Figure 2B). Regulation of H6PDH mRNA expression showed a similar profile to 11β‐HSD1, being elevated in response to DEX (*P* < .05) and DEX combined with insulin (*P* < .05; Figure 2C). Glucose‐6‐phosphate transporter mRNA did not deviate from control levels with any treatments (Figure 2D). Thus, glucocorticoid can positively regulate 11B‐HSD1 and H6PDH expression and result in increased 11β‐HSD1 activity in differentiated myotubes.

### 3.3 Muscle‐specific deletion of 1β‐hydroxysteroid dehydrogenase type 1

To investigate the muscle‐specific roles of 11β‐HSD1 in the context of whole body metabolic homeostasis and in response to glucocorticoid excess, we developed muscle‐specific HSD1MKO mice. The generation and utilization of *HSD11B1* floxed mice have been previously described and used extensively to investigate global, hepatocyte, and adipocyte loss of function.^14^, ^16^, ^17^, ^18^ Initially, we evaluated the MEF2c‐Cre transgenic, but this did not result in Cre expression and failed to reveal recombination of the floxed *HSD11B1* locus or effects on 11β‐HSD1 activity, and as such, this line was abandoned. We then evaluated 2 additional Cre lines against the floxed *HSD11B1* allele‐MCK‐Cre and ACTA1‐Cre. Both transgenics expressed Cre and resulted in high level recombination of the floxed *HSD11B1* to a null allele. As MCK‐Cre and ACTA‐Cre generated identical effects on 11β‐HSD1, for clarity, we present the data associated with ACTA1‐Cre‐ *HSD11B1* cross.

**Figure 3:**
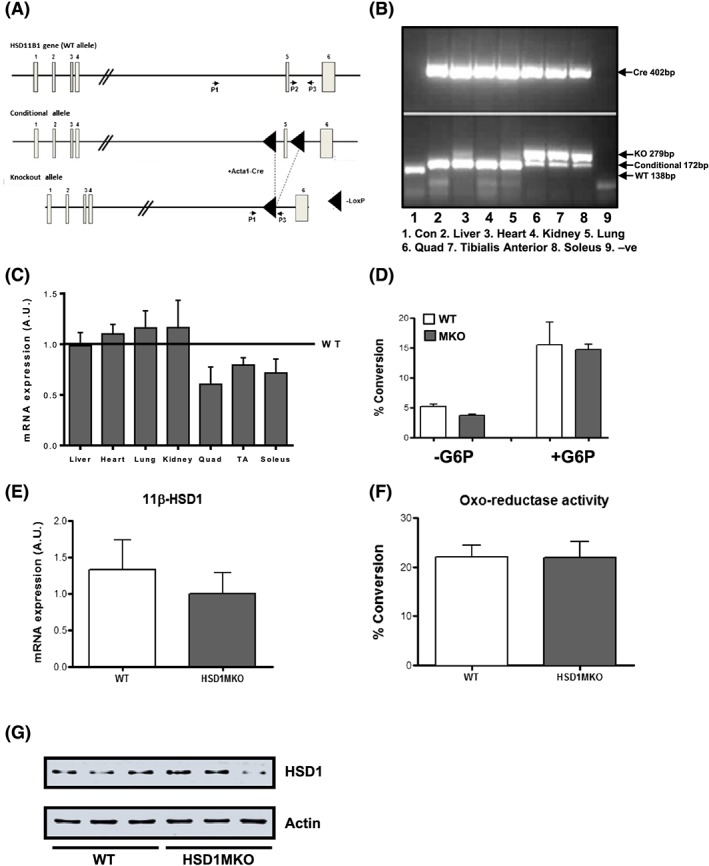
Strategy and evaluation of muscle‐specific 11β‐HSD1 knockout (HSD1MKO) A, Targeting strategy for HSD1MKO generation. Exon 5 of the conditional *HSD11B1* allele is flanked with LoxP sites and bred with the muscle‐specific Acta1‐Cre mice to generate HSD1MKO knockout allele mice. B, Polymerase chain reaction (PCR) detection of Cre positivity and a knockout allele only in muscle tissues, validating the HSD1MKO model. The PCR products were derived from HSD1 primer sets on genomic DNA isolated from a range of muscle and nonmuscle tissues. C, 11β‐Hydroxysteroid dehydrogenase type 1 (11β‐HSD1) mRNA expression measured in tissue from HSD1MKO and wild‐type (WT) mice, with WT levels set at 1 across tissues. D, 11β‐HSD1 oxo‐reductase activity in muscle‐derived microsomes, with or without 10 mM G6P, from HSD1MKO and WT mice. E, 11β‐HSD1 mRNA expression and F, 11β‐HSD1 oxo‐reductase activity in primary myoblasts derived from HSD1MKO and WT mice. G, Immunoblot of 11β‐HSD1 protein in HSD1MKO and WT quadriceps muscle (*n* = 6/group *P < .05, **P < .01, ***P < .001 versus WT; Quad, quadriceps; TA, tibialis anterior)

Mice homozygous for floxed *HSD11B1* alleles were crossed with mice heterozygous for a floxed allele and hemizygous for the Acta1‐Cre transgene expressing Cre recombinase under the control of *Acta1* (alpha actin) promoter (Figure 3A). Recombination between LoxP sites (removal of exon 5) generated KO alleles restricted to skeletal muscle as assessed in quadriceps, soleus, and tibialis anterior (TA). All other tissues assessed—including liver, lung, kidney, and heart (although it expresses *Acta1* gene)—remained normal for conditional floxed exon 5 *HSD11B1* alleles (Figure 3B). We next assessed levels of 11β‐HSD1 mRNA by real‐time PCR by using primers and probes positioned to detect cDNA sequences corresponding to exon 5 in liver, heart, lung, kidney, quadriceps, soleus, and TA tissues. Levels in HSD1MKO tissues were compared with the same tissue collected from control mice (negative for the Cre transgene; Figure 3C). While there were clear reductions in mRNA expression in muscle tissues (30‐40%), they were not commensurate with the levels anticipated given the degree of genomic recombination. Indeed, in liver and adipocyte 11β‐HSD1KO mice, the same level of genomic recombination seen here was then mirrored by near complete loss of mRNA and enzyme activity.^14^, ^16^, ^18^ To further examine this model, the oxo‐reductase activity of 11β‐HSD1 in the SR was measured in intact microsomes prepared from skeletal muscles. 11β‐Hydroxysteroid dehydrogenase type 1 reductase activity was 3‐fold stimulated in microsomes preincubated with 10 mM G6PT as previously shown.^10^ However, there was a similar level of induction of activity in muscle microsomes from HSD1MKO, corroborating the mRNA data (Figure 3D). 11β‐Hydroxysteroid dehydrogenase type 1 expression and activity were also measured in primary cultured WT and HSD1MKO myotubes (Figure 3E and F). Again, although there was a decrement in the level of mRNA in HSD1MKO myotubes, there was no difference between HSD1MKO and WT cells in the ability to reduce 11‐DHC to corticosterone (Figure 3E and F). Finally, we immunoblotted for 11β‐HSD1 protein in lysates of quadriceps from control and HSD1MKO mice and showed that protein levels were similar in all animals tested, irrespective of genotype (Figure 3G). Thus, while the HSD1MKO model was technically successful in achieving high level, muscle‐restricted, recombination of the *HSD11B1* conditional allele to a KO allele, it appears insufficient to impair 11β‐HSD1 protein expression or enzyme activity.

### 3.4 Muscle‐specific deletion of hexose‐6‐phosphate dehydrogenase

**Figure 4:**
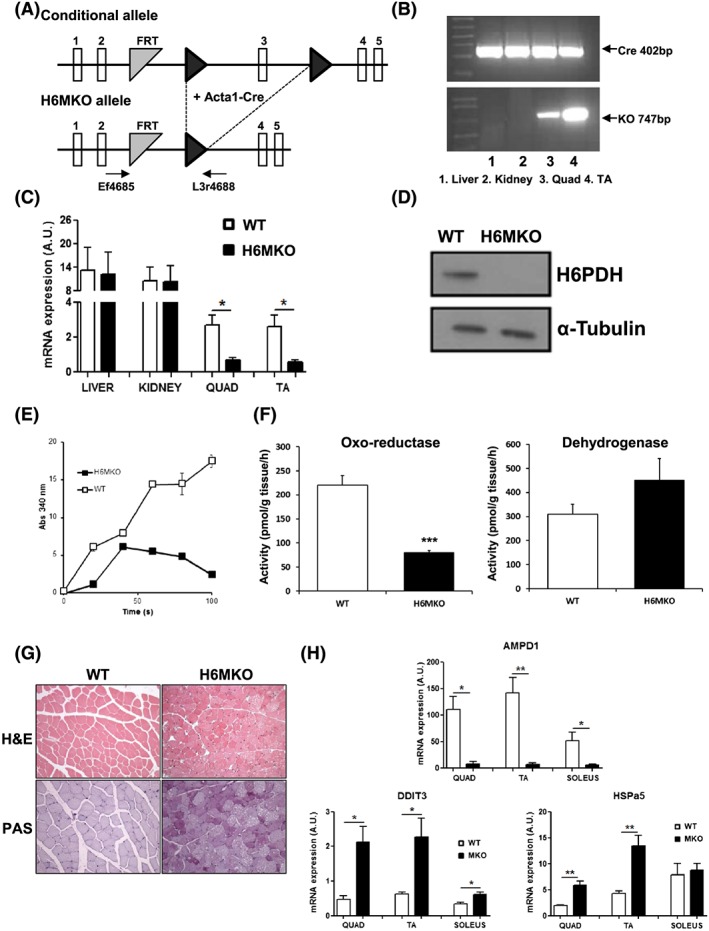
Strategy and evaluation of hexose‐6‐phosphate dehydrogenase (H6MKO) A, Targeting strategy for H6MKO generation. Exon 3 of a conditional allele (purchased from the European Conditional Mouse Mutagenesis Program consortium) flanked with LoxP sites and bred with the muscle‐specific Acta1‐Cre mice to generate H6MKO knockout allele mice. B, Polymerase chain reaction (PCR) detection of Cre positivity and a knockout *H6PD* allele only in muscle tissues, validating the H6MKO model. The PCR products were derived from H6PD primer sets on genomic DNA isolated from muscle and nonmuscle tissues. C, Real‐time PCR analysis of relative H6PDH mRNA expression in tissues from H6MKO mice. D, Immunoblot analysis of H6PDH protein expression in quadriceps from H6MKO mice. E, Generation of NADPH from NADP by H6PDH in microsomes isolated from H6MKO muscle. F, 11β‐Hydroxysteroid dehydrogenase type 1 (11β‐HSD1) oxo‐reductase activity and corresponding dehydrogenase activity in quadriceps muscle explants from wild type (WT) and H6MKO mice. G, Histological analysis by haematoxylin and eosin (H&E) and periodic acid Schiff (PAS) staining (for glycogen content) in WT and H6MKO quadriceps tissue. H, AMPD1 and ER stress markers DDIT3 and HSPa5 mRNA expression in quadriceps, TA, and soleus muscle from WT and H6MKO mice (*n* = 6/group *P < .05, **P < .01, ***P < 0.001 versus WT; Quad, quadriceps; TA, tibialis anterior)

To allow investigation of the muscle‐specific roles of H6PDH in the context of whole body metabolic homeostasis, we have developed muscle‐specific H6MKO mice. Mice homozygous for a conditional floxed exon 3 *H6PD* allele were crossed with mice heterozygous for a floxed allele and hemizygote for the Acta1‐Cre transgene (Figure 4A). Initial characterization of H6MKO mice by using genomic DNA isolated from a range of tissues showed that recombination of the conditional allele to the KO allele is skeletal muscle‐specific (quadriceps and TA), with other tissues (including liver and kidney) remaining unaltered (Figure 4B). Hexose‐6‐phosphate dehydrogenase mRNA was assessed in the quadriceps and TA muscles of control and H6MKO mice by real‐time PCR and reduced by approximately 75% in the H6MKO compared with WT mice (Figure 4C). Hexose‐6‐phosphate dehydrogenase protein was almost undetectable when assessed by immunoblot of lysates prepared from quadriceps of H6MKO and control mice (Figure 4D). Using microsomes prepared form quadriceps, we also show almost no ability to generate NADPH from NADP+ in the H6MKO mice by using a H6PDH‐specific assay (Figure 4E). Previously, we have shown that 11β‐HSD1 activity in muscle explants of global H6PDHKO mice changes from a reductase to a dehydrogenase.^10^, ^19^ We measured these 11β‐HSD1 activities in quadriceps muscle of H6MKO and show a significant loss of oxo‐reductase activity, with a nonsignificant change in dehydrogenase activity, largely endorsing the anticipated 11β‐HSD1 biochemistry (Figure 4F). Muscle of H6PDHKO mice displays a type IIb fibre vacuolated myopathy and elevated glycogen storage.^19^ We assessed the histological features of control and H6MKO quadriceps by haematoxylin and eosin and periodic acid‐Schiff staining for glycogen content (Figure 4G). This analysis again corroborated the muscle architecture and increased glycogen storage consistent with a loss of muscle H6PDH activity (Figure 4G).

Finally, profiling of muscle from global H6PDHKO mice revealed a distinct pattern of metabolic genes dysregulated in their expression and coordinate activation of genes of the ER stress and unfolded protein responses.^19^ We measured a number of these signature genes in H6MKO quadriceps, TA, and soleus and show H6MKO muscle to mirror global H6PDHKO, here illustrated by reduced AMPD1 (most downregulated metabolic gene) and increased HSPa5 (ER stress sensor) and DDIT3 (ER stress effector) in H6MKO compared with controls (Figure 4H). As previously seen, the effects on soleus in ER stress response are minimal or absent (ie, HSPa5). Thus, unlike the HSD1MKO mice, we show that H6PDH was significantly reduced in skeletal muscle, leading to a muscle‐specific phenocopy of the myopathy found previously in global H6PDHKO mice.

## 4 DISCUSSION

Intracellular glucocorticoid generation in skeletal muscle relies on the coordinate expression of the proteins G6PT/H6PDH/11β‐HSD1. Hexose‐6‐phosphate dehydrogenase and G6PT expressions are fairly constant during postmuscle cell differentiation, suggesting that their levels are sufficient to facilitate maintenance of appropriate SR redox conditions, whereas 11β‐HSD1 expression and activity increase over differentiation and are further responsive to exogenous glucocorticoids.

11β‐Hydroxysteroid dehydrogenase type 1 expression is regulated by a plethora of hormones and cytokines, acting to increase or decrease the ability of target cells to generate glucocorticoid.^5^ Glucocorticoids are known to increase the expression and activity of 11β‐HSD1, which may be an important physiological mechanism to locally increase available glucocorticoid and restrain the acute inflammatory process, a response potentially perturbed in disease.^20^, ^21^ We show here that glucocorticoid signalling increases the expression of 11β‐HSD1 and H6PDH, but not G6PT in muscle. In the context of endogenous or exogenous glucocorticoid excess, upregulation of 11β‐HSD1 in muscle may represent an unwanted “side‐effect” exacerbating myopathy.^3^, ^16^ In support of this, 11β‐HSD1KO mice do not display detectable defects in muscle physiology indicating that 11β‐HSD1 is dispensable for differentiation and more critical to modulating muscle tissue responses to glucocorticoid. However, 11β‐HSD1KO mice are protected from the adverse metabolic effects of glucocorticoid excess, with muscle being particularly resistant.^16^ Thus, we have established that regulation of 11β‐HSD1 expression, more so than H6PDH or G6PT, appears a robust mechanism of altering intracellular capacity to regenerate glucocorticoid and mechanistically linked to propagating the deleterious effect of glucocorticoid excess in muscle.^3^, ^16^

We propose that muscle 11β‐HSD1 expression can modify global metabolic phenotype in response to elevated GC availability and is therefore a legitimate therapeutic target tissue in the context of disease, and selective 11β‐HSD1 inhibitors are a promising therapy to limit the side‐effects associated with GC excess. Furthermore, it has been shown that expression and activity of 11β‐HSD1 are elevated in skeletal muscle of diabetic individuals and pharmacological inhibition of 11β‐HSD1 is insulin sensitizing.^12^, ^22^

To assess these notions in more detail, we established 11β‐HSD1 and H6PDH muscle‐specific KO models, in which we would be able to examine global and tissue‐specific phenotypes in the context of metabolic disease such as glucocorticoid excess. Although we successfully generated HSD1MKO mice with high‐level genomic recombination at the *HSD11B1* locus, to our surprise, robust 11β‐HSD1 activity was present in skeletal muscle tissues. This was in contrast to highly efficient knockout of 11β‐HSD1 activity restricted to liver and adipose.^14^, ^16^ Although the reason for this result is not entirely clear, we can propose a likely scenario for these observations. ACTA1‐Cre‐mediated recombination is never 100% efficient in any tissue or cell type, and in the case of Acta‐Cre, up to 10% of alleles remain unrecombined, essentially retaining the potential for WT gene expression.^23^ Muscle is a multinucleate cell‐type, and therefore, unrecombined *HSD11B1* alleles could produce sufficient mRNA and protein to retain almost control levels of 11β‐HSD1 reductase activity, hence an inability to measure meaningful differences. We propose that, in a stable system and at any one time, the rate of functional 11β‐HSD1 accumulated from unrecombined alleles is greater than the rate of clearance through proteasomal degradation, and so the tissue can essentially retain WT levels of activity. In support of this, previous reports have suggested a long protein half‐life for 11β‐HSD1 leading to persistence of protein, and thus enzyme activity, in a range of cell types and models using similar genetic knockout or knockdown strategies.^24^, ^25^, ^26^ However, this remains to be formally tested in the context of HSD1MKO mice. Similarly, we have not gone on to test HSD1MKO mice against glucocorticoid excess. It may be that the unrecombined alleles are unable to achieve the levels of 11β‐HSD1 induction that would be seen in WT mice; hence, there would be reduced intracellular glucocorticoid regenerative capacity and a protective effect would emerge.

We and others have previously shown that global H6PDH deficiency causes metabolic defects associated with changes to 11β‐HSD1‐mediated glucocorticoid metabolism in tissues such as liver and adipose tissue. However, myopathy seen in global H6PDH deficiency is glucocorticoid and 11β‐HSD1 independent, but it was unknown whether loss of H6PDH in nonskeletal muscle tissue contributed to myopathy.^17^ Having now generated and validated H6MKO mice, we can confirm phenocopy of the myopathy seen in global H6PD KO mice and conclude that the phenotype originates in muscle and is not influenced by other tissues. It would appear that, unlike HSD1MKO mice, H6PDH is a locus more susceptible to complete recombination, substantially reducing protein levels insufficient to support 11β‐HSD1 activity. This model will be useful for understanding how skeletal muscle‐specific metabolic perturbation due to H6PDH SR loss affects global metabolic homeostasis and as a model to evaluate muscle specific responses to glucocorticoid excess.

## CONFLICT OF INTEREST

The authors have declared that there is no conflict of interest.
